# Identification of human pathogens in soil by virulence gene-based machine learning method

**DOI:** 10.1016/j.eehl.2025.100171

**Published:** 2025-07-24

**Authors:** Shengchun Qi, Shuyan Wang, Yu Xia, Songcan Chen, Huijie Lu

**Affiliations:** a State Key Laboratory of Soil Pollution Control and Safety, Zhejiang University, Hangzhou 310058, China; b Laboratory of Environmental Microbiology and Ecological Genomics, College of Environmental Science and Engineering, Southern University of Science and Technology, Shenzhen 518055, China; c Key Laboratory of Environment Remediation and Ecological Health, Ministry of Education, College of Environmental Resource Sciences, Zhejiang University, Hangzhou 310058, China

**Keywords:** Virulence factor, Human pathogenic bacteria, Soil, Metagenome, Machine learning

## Abstract

Soils are important reservoirs of human pathogenic bacteria that can spread to humans through various pathways. Metagenomics enables high-throughput pathogen identification by mapping sequencing reads to known pathogen genomes. However, this approach has several limitations, e.g., sequence assembly is time-consuming, and reliance on reference databases may overlook potential pathogens lacking close genomic matches. Here, we developed a novel, virulence factor (VF) based machine learning method using the K-Nearest Neighbors model (VF–KNN) for identifying human pathogenic bacteria from soil metagenomes. Through learning the VF features of pathogenic and non-pathogenic bacteria, VF-KNN could achieve the desired performance in soil pathogen identification (AUC: 0.95, Accuracy: 0.85). Model prediction accuracy (0.95) was further validated using 61 pathogenic strains isolated from soil. For the top 15 most frequent soil pathogens, the prediction accuracy was >0.90 ​at 0.4X–1.0X genome coverage. VFs contributing significantly to pathogen identification were associated with regulation, effector delivery, motility, etc. By using VF-KNN, the averaged abundance of total potential pathogens in topsoils across China was 0.44% (*n* ​= ​336), predominantly concentrated in the eastern coastal provinces. Compared with the conventional method based on a predefined pathogen list, VF-KNN identified 28% more potential pathogenic species, including some newly reported but not in the predefined list (e.g., *Mycolicibacterium cosmeticum*). Agricultural land exhibited significantly higher pathogen abundance and diversity than the other land types. This newly developed VF-KNN method is applicable for pathogen identification in broader environments.

## Highlights

• VF-KNN enables identification of potential human pathogens in soil metagenome with high accuracy, sensitivity, and biological interpretability.
• VFs related to regulation and mobility strongly affect pathogen identification by VF-KNN.
• VF-KNN identifies more soil pathogenic species than Patholst, including potentially new pathogens, e.g., *Mycolicibacterium cosmeticum.*
• Agricultural land has higher pathogen abundance and diversity than other land types across China.

## Graphical abstract

**Figure:**
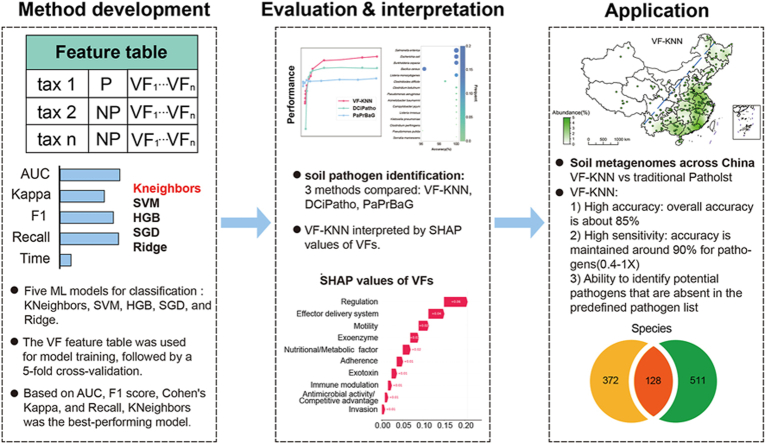

## 1 Introduction

Soils are important reservoirs of diverse human bacterial pathogens that pose threats to public health through exposure, inhalation, and consumption of agricultural and livestock products [1]. Common bacterial pathogens in soils include *Escherichia coli*, *Salmonella, Bacillus anthracis*, and *Pseudomonas aeruginosa,* with abundances spanning from 10 to 10^5^ ​CFU/g dry soil [[2], [3], [4]]. Although soil bacterial pathogens are generally less abundant than those in other reservoirs, e.g., animals and water, their levels can rise significantly due to fecal contamination [5]. In recent years, climate change, environmental pollution, and global mobility have profound impacts on pathogenic bacteria, contributing to increased incidence of soil-borne diseases, such as Lyme disease, leptospirosis, and tularaemia [6,7]. This underscores the urgent need for accurate and rapid identification of pathogenic bacteria in soils [8].

Traditional culture-based pathogen identification methods involve cultivating microorganisms on selective media, followed by phenotypic characterization and immunological assays [9]. These methods are time-consuming, low-throughput, and limited by the number of culturable microorganisms [10,11]. PCR assays have higher sensitivity, specificity, and speed for pathogen identification compared to culture-based methods [12]. However, they are unable to identify potentially new pathogens and have limited throughput. Next-generation sequencing (NGS) technologies and the associated computational tools enable high-throughput pathogen identification with high speed, sensitivity, and resolution [13,14], facilitating the comprehensive understanding of pathogenic bacteria and how they interact with their surrounding environments [15].

Pathogen identification methods can be broadly categorized into protein-based and whole genome-based [16]. Protein-based methods such as PaPrBaG and BacFier rely on protein profiles or the identification of virulence factors (VFs) to determine bacterial pathogenicity [16,17]. Whole genome-based methods, e.g., DCiPatho, Kraken, and MetaPhlAn, use either k-mer features or evolutionary branch-specific marker genes to identify pathogens in microbial communities [[18], [19], [20]]. They primarily focus on taxonomic classification and comparison with known pathogens, and a large number of reads that fail to align with reference genomes are usually discarded, including those that belong to low-abundance pathogens, leading to underestimation.

We propose an innovative approach that integrates VF features in pathogen classification based on machine learning (ML) algorithms. VFs are present in both pathogens and non-pathogens; however, their VFs composition patterns (e.g., gene combinations, genomic contexts) differ significantly. For example, *Rickettsia conorii* causing the life-threatening Mediterranean spotted fever and *Rickettsia montanensis* that has limited or no pathogenicity are substantially different in their VFs related to Type IV secretion, immune evasion, and replication [21]. This distinction forms the theoretical foundation for developing a machine learning-based approach for pathogen identification. In this study, we compared the performance of multiple algorithms for pathogen/nonpathogen classification and selected the best-performing K-Nearest Neighbors (VF–KNN). VFs that strongly influence pathogen identification were determined. VF-KNN was subsequently employed to analyze bacterial pathogens in topsoil metagenomes across China, yielding new insights into their geographic distribution, land-type impacts, and correlations with soil properties. VF-KNN is broadly applicable to pathogen identification based on environmental or even clinical metagenomes. It also provides valuable guidance for developing specific VF biomarkers that allow rapid monitoring of pathogens in the environment.

## 2 Materials and methods

### 2.1 Dataset preparation

#### 2.1.1 Dataset 1

A total of 18,941 complete bacterial genomes were downloaded from the National Center for Biotechnology Information (NCBI, https://www.ncbi.nlm.nih.gov/refseq). Among them, 12,885 genomes were tagged as “pathogenic” (belonging to 611 bacterial pathogen species), and the other 6056 were tagged as “non-pathogenic” (3355 species), following the labeling procedure as per [19]. Although non-pathogenic genomes exhibit far greater abundance and diversity in the environment, their utility for model training remains severely limited due to the scarcity of extractable virulence gene information. Therefore, although we initially included more non-pathogenic genomes, only those with extractable virulence gene information were eventually used in model training. In fact, creating an imbalanced Dataset 1 and ensuring pathogens are more abundant than non-pathogens makes the machine learning process more targeted, reducing the risk of misclassifying non-pathogens that carry VF information as pathogens.

In order to simulate the characteristics of pathogenic bacteria reads produced by second-generation sequencing, the above genomes were modeled into an artificial metagenome, with a fold coverage of 1X using the ART illumina simulator [22] with necessary trimming and filtering steps. The read length was 150 bp, the mean size of DNA fragments for paired-end simulations was 500 bp, the standard deviation of DNA fragment size for paired-end simulations was 10 bp, and the raw quality profile was HS25.

#### 2.1.2 Dataset 2

4130 genomes were downloaded from the NCBI Pathogen Detection Program database with “isolation_source: ∗soil∗”, and tagged as “pathogenic” (135 species). Another 3046 genomes were tagged as “non-pathogenic” (2261 species), following the labeling procedure as per [19]. These data were also simulated into an artificial metagenome, with 13-fold coverage gradients ranging from 0.001X to 1X by the ART simulator, with the same parameters as Dataset 1.

Detailed information of Dataset 1 and 2 can be found in Supplementary Data 1.

### 2.2 VF-KNN method development

**Fig. 1:**
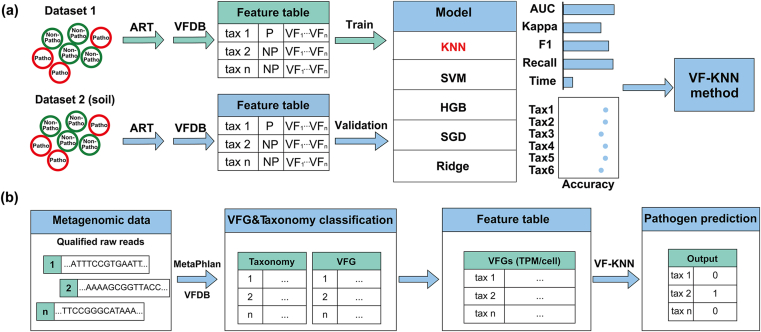
Overview of the VF-KNN method for pathogen identification. (a) VF-KNN method development. The VF features of pathogens and nonpathogens in Dataset 1 were used for training, testing, and validation, and those in Dataset 2 were used for external validation. Five ML algorithms were compared in terms of AUC, Kappa, F1, Recall, and the prediction time of pathogen identification. (b) VF-KNN workflow for pathogen identification based on metagenomic data.

Based on Dataset 1, the taxonomic information was resolved to the strain level, and VF genes were annotated according to the latest virulence factor database (VFDB) [23] (Fig. 1a). A comprehensive VF feature table was created for both pathogens and nonpathogens. The VF features were expressed as transcripts per million per cell (TPM/cell), where the cell number was estimated by mapping against an essential single-copy marker gene database using ARG-OAP [24,25]. TPM values were calculated using Eqs. (1), (2) as per [26]. The VF features for each strain included the TPM/cell values for total, category-summarized (14 categories), and individual VFs (565 types).

(1) $$rpk=\frac{n_{\mathrm{VF}}\times n_{\mathrm{nt}}}{n_{\mathrm{VF}-\mathrm{nt}}}$$

(2) $$TPM=\frac{rpk\times 10^{6}}{\sum rpk}$$

where, *rpk*: VF reads per kilobase; *n*_VF_: the number of reads mapped to a particular VF gene; *n*_nt_: the average number of nucleotides mapped per read; *n*_VF-nt_: the number of nucleotides in a mappable region of a VF gene.

Five ML algorithms for classification were selected, including KNN, Support Vector Machines (SVMs), Histogram-based Gradient Boosting (HGB), Stochastic Gradient Boosting (SGD), and Ridge. The VF feature tables were used for model training, followed by a 5-fold cross-validation. In each fold, 67.5% of the data were utilized for training, 22.5% for testing, and 10% for validation. The five models were mainly compared using performance metrics, including area under the ROC curve (AUC, equivalent to the probability that the classifier will rank a randomly chosen positive instance higher than a randomly chosen negative instance) [27], F1 score, Cohen's Kappa, and Recall. Additional performance metrics, including precision, accuracy, precision–recall curves (PRC), and receiver operating characteristic (ROC) curves, were also analyzed.

Dataset 2 was used as external validation to further evaluate the performance of the five models in analyzing soil metagenomes. Model performance was measured by a series of parameters, including AUC, F1 score, Cohen's Kappa, recall, time of prediction, accuracy, precision, precision-recall, PRC, and ROC. The KNN model was selected for developing the VF-KNN method.

In VF-KNN, sequences with relative abundances less than 0.001% were discarded (noise), and thus, pathogen/non-pathogen identification cannot be conducted for species with extremely low abundance. To improve model performance and potentially reduce false positives or misclassifications, the model hyperparameters, e.g., n*_*neighbors, were further optimized for VF-KNN. Detailed feature scaling and hyperparameter tuning procedure and results can be found in Supplementary Information (Fig. S7). Key parameters after hyperparameter optimization were: the number of neighbors was 10, the weight function used in prediction was set as “distance”, and the power parameter for the Minkowski metric was 1 (equivalent to manhattan_distance).

### 2.3 Comparisons between VF-KNN and other pathogen identification methods

In order to benchmark our method against existing methods, VF-KNN was compared with PaPrBaG [16] and DCiPatho [19] using Dataset 2. PaPrBaG is based on a random forest algorithm for training and identifying pathogenic bacteria based on sequence k-mer and protein features. DCiPatho employs deep cross-fusion networks to identify pathogenic bacteria based on sequence k-mers. Their performance was evaluated, including AUC, F1 score, Cohen's Kappa, Recall, Accuracy, and Precision. Score-coverage curves were used to measure the sensitivity of different methods as a function of genome fold coverage (0.001X–1.0X).

To further evaluate the impacts of different VFs on pathogen prediction, the SHapley Additive exPlanations (SHAP) values were calculated for each VF using the Shap package in Python [28].

### 2.4 VF-KNN workflow for pathogen identification based on metagenomic data

The VF-KNN workflow started from quality control of metagenomic reads using Fastp (Fig. 1b). Taxonomy classification was performed using MetaPhlAn at the strain level. VFs annotation, TPM/cell calculation, and feature table construction (including both taxonomy and VFs information) were conducted as described in *Section* 2.2. The comprehensive feature table was used as input data for the VF-KNN method. As a result, metagenome assembly can be avoided, and VF information can be tightly correlated with taxonomy information to obtain better pathogen prediction.

### 2.5 Analysis of human pathogens in topsoils across China

Metagenomic data (about 17 GB/sample) of 336 topsoil samples across China were downloaded from NCBI. Samples were classified into five land types, including farmland, forest, grassland, wetland, and artificial surface (i.e., soils in areas such as streets and the surroundings of buildings) as per [29]. Soil properties of the 336 samples were obtained from the 1:1 million soil data provided by the Institute of Soil Science, Chinese Academy of Sciences, sourced from the Second National Land Survey (from the HWSD version 1.2), including soil texture, pH, bulk density, cation exchange capacity (CEC), exchangeable sodium percentage (ESP), and so on. Information on the 336 SRA files and soil properties can be found in Supplementary Data 2.

Bacterial pathogens were analyzed using both VF-KNN and the conventional method based on a predefined pathogen list (named Patholst) [19] derived from 3464 pathogen genomes. All SRA files were split into paired-end raw reads using fastq-dump (v3.1.1) in sratoolkit (v3.1.1) with the option “split-3”. The quality control was then performed using fastp v0.23.4. Taxonomy classification was carried out using MetaPhlAn v4.0.6 ​at the strain level. For Patholst, pathogens were identified by comparing them with the pathogen list. For VF-KNN, VF genes were further annotated using blastn (v2.15.0) and VFDB (v2024.01.23). The comprehensive VFs feature table was constructed as the input for VF-KNN, and all taxa were identified as pathogens or non-pathogens.

The alpha and beta diversity of pathogens at the species level were determined using the R package vegan (v2.6-8). The abundance and composition of pathogens, as well as the 14 categories of VFs, were compared for soils belonging to the five land types. Co-occurrence network analysis was performed for VFs and pathogens, and plotted using Gephi. Correlations between total pathogen abundance, alpha diversity, and VFs abundance in 14 categories with representative soil physicochemical properties were indicated by Spearman's correlation coefficients.

## 3 Results

### 3.1 KNN outperforms other ML models in pathogen prediction

**Fig. 2:**
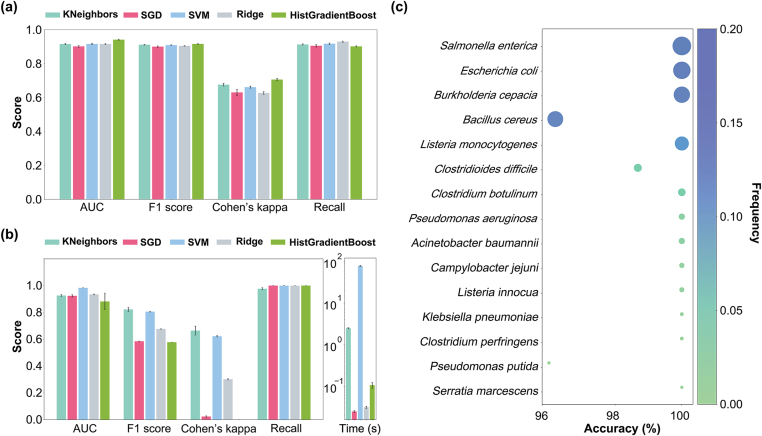
Performance evaluation of five ML models. (a) AUC, Cohen's Kappa, F1 score, and Recall values of the five models on Dataset 1; (b) AUC, Cohen's Kappa, F1 score, Recall value, and Prediction time of the five models on Dataset 2 (fold coverage: 1X); (c) Prediction accuracy for the top 15 frequent pathogens in Dataset 2 (fold coverage: 1X) using the VF-KNN method. The 15 pathogens are ranked according to their frequencies in Dataset 2, which are represented by the size and color of the bubbles.

The five ML models performed almost equally well on Dataset 1, with AUC 0.90–0.94, F1 0.90–0.92, Cohen's Kappa 0.63–0.71, and Recall 0.90–0.93 (Fig. 2a). However, on the validation Dataset 2 (source: soil, genome fold coverage: 1X), all four scores were reduced, especially Cohen's Kappa (Fig. 2b). KNN and SVM still achieved relatively high Cohen's Kappa values (>0.6) [30], while the values of the other three models were close to 0. In general, Gradient Boosting can outperform KNN, particularly for high-dimensional feature spaces or non-linear relationships. However, the GradientBoost method demonstrated poor generalization ability on Dataset 2. This may be due to the relatively small size of the training Dataset 1 and the complexity of the GB model, which can easily lead to overfitting.

We further compared the prediction time of the five models on Dataset 2 under identical hardware conditions. The computer infrastructure can be found in the Supplementary Information. The SVM model had the longest prediction time of 78.68 ​s, followed by KNN (2.49 ​s), and the remaining models all completed predictions within 1 ​s (Fig. 2b). Additional performance indices of different models on Datasets 1 and 2 can be found in Figs. S1 and S2.

These results indicate that KNN not only demonstrates excellent performance and generalization ability but also maintains high computational efficiency on Dataset 2, making it a highly promising tool for identifying pathogenic bacteria based on soil metagenomic data. Therefore, we selected KNN as the best-performing ML algorithm, and the corresponding method was named VF-KNN. VF-KNN represents a lightweight and high-performance option when applied to relatively large metagenome Datasets.

The prediction accuracy (true positive rate) of the VF-KNN model for the top 15 frequent soil pathogens was assessed. These pathogens constitute 97% of all pathogens in Dataset 2, including *Salmonella enterica*, *E*. *coli*, and *Burkholderia cepacia*, and so on. Their prediction accuracy by using VF-KNN ranged from 96% to 100% at genome coverage of 1X (Fig. 2c).

To demonstrate the reliability of VF-KNN for predicting pathogenic bacteria in real soil samples, we further isolated and sequenced the whole genomes of 61 pathogenic bacteria from soil, followed by serological pathogenicity assays. The VF-KNN method demonstrated 95% prediction accuracy for these pathogenic isolates. Detailed procedures of bacteria isolation and serological assays were presented in Table S1.

### 3.2 VF-KNN achieved the desired performance on soil pathogen identification

**Fig. 3:**
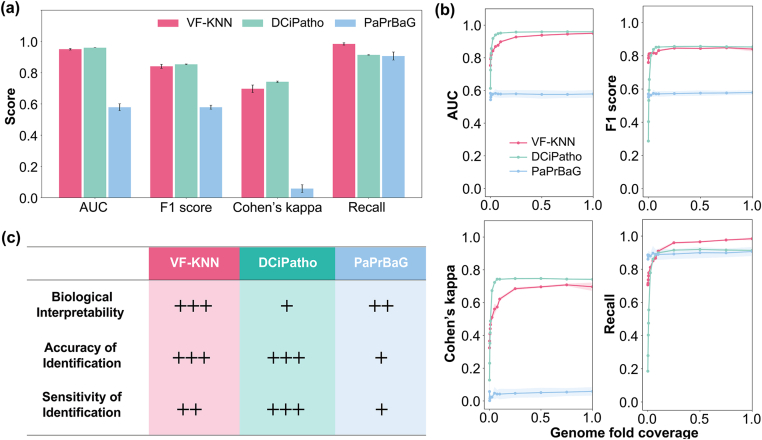
(a) Performance of VF-KNN, DCiPatho, and PaPrBaG on Dataset 2; (b) Performance of the three methods as a function of genome fold coverage; (c) Comparisons between the three methods.

After hyperparameter optimization, VF-KNN was compared with two specialized pathogen identification methods, namely DCiPatho and PaPrBaG, on their performance using Dataset 2 (Fig. 3a). The overall performance of VF-KNN was comparable to that of DCiPatho and was significantly higher than that of PaPrBaG. Specifically, the Cohen's Kappa value for VF-KNN was 11-fold higher than that of PaPrBaG. For all three methods, the AUC, F1 score, Cohen's Kappa, and Recall values increased as the genome fold coverage increased, reaching a plateau around 0.4X (Figs. 3b and S3). However, the plateau values of PaPrBaG were lower than those of the other two methods. VF-KNN method achieved desired performance, i.e., AUC 0.95, F1 0.84, Cohen's Kappa 0.70, Recall 0.98, Accuracy 0.85, Precision 0.73. Although it was slightly inferior to DCiPatho (AUC, F1 score, and Cohen's Kappa were about 20% lower, at fold coverage of 0.4X), its Recall value was about 25% higher than that of DCiPatho. The VF-KNN model (n_neighbors ​= ​10) achieved an average false positive rate of 8.7% and an average true positive rate (recall) of 95.7% on Dataset 1. On Dataset 2, the false positive rate was 23.6%, while the true positive rate reached 99.3%. Comparatively, the false positive rate of PaPrBaG was 83.8%. The relationships between true and false positive rates were illustrated by the ROC curves in Figs. S1 and S2.

Additional performance indices of the three methods on Dataset 2 can be found in Fig. S3. The comparisons of the three methods are briefly summarized in Fig. 3c, and more details can be found in Table S2. Overall, VF-KNN demonstrates desired accuracy and sensitivity even under relatively low genome coverage (0.4X–1X, Fig. S4).

For the top 15 frequent pathogens in Dataset 2, prediction accuracy was further compared for the three methods (Fig. S4). The prediction accuracy of VF-KNN was comparable to that of DCiPatho, but higher than PaPrBaG. Specifically, for the top three frequent pathogens in Dataset 2, namely *S*. *enterica*, *E*. *coli*, and *B*. *cepacia*, VF-KNN achieved higher prediction accuracy (98%–100%) than DCiPatho (67%–100%) and PaPrBaG (59%–100%). The standard deviations of prediction accuracy of DCiPatho and VF-KNN were also lower than that of PaPrBaG. At a relatively low genome fold coverage of 0.4X, VF-KNN could achieve the desired overall prediction accuracy of >90%.

### 3.3 Impacts of VFs on pathogen identification

**Fig. 4:**
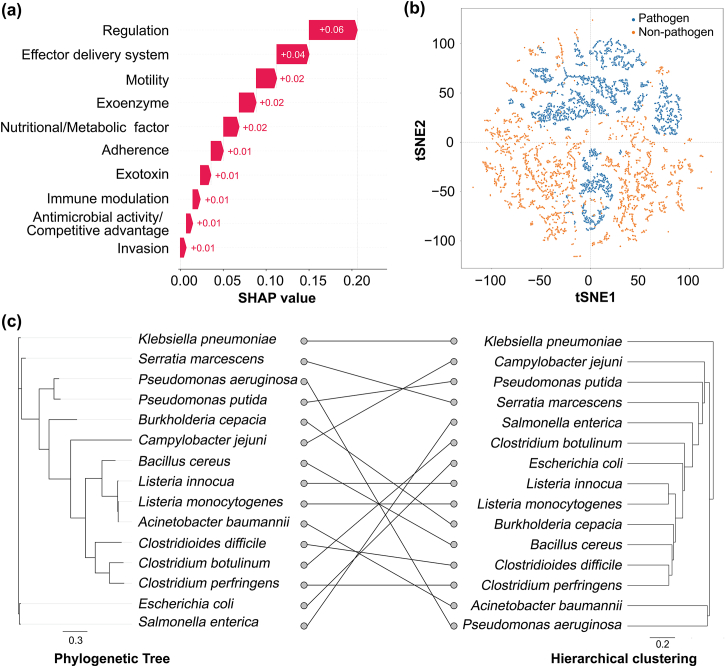
Interpretation of the VF-KNN method. (a) Mean SHAP values of the top 10 VF categories influencing pathogen identification. Numbers are the summed SHAP values of VFs within each category; (b) t-SNE non-linear dimensionality reduction applied to pathogens and non-pathogens in Dataset 2 (1X) based on their VF features; (c) Hierarchical clustering tree and phylogenetic tree of the top 15 frequent pathogens in Dataset 2. Lines between trees link the same pathogenic species, and crossing lines indicate a lack of similarity in the two trees.

The interpretability of the VF-KNN method is closely tied to VF features. We calculated the impacts (SHAP values) of all VFs based on 200 genomes randomly selected from Dataset 2. SHAP values essentially measure how much each VF influences the classification results, with higher values indicating stronger impacts on pathogen identification. For each VF, we computed its SHAP value and summed the SHAP values of all VFs within the same category to assess the overall contributions of each VF category to pathogen prediction (Fig. 4a). VFs with relatively strong impacts on pathogen prediction were associated with regulation, effector delivery system, motility, exoenzyme, and metabolic factor, etc., and those strongly influencing nonpathogen prediction were listed in Fig. S5. We further performed tSNE analysis on pathogens and nonpathogens in Dataset 2 (Fig. 4b), and observed a separation between the two groups. This further supports the validity and reliability of using VFs features for pathogen prediction.

We observed pathogen-specific VF SHAP profiles, e.g., *Klebsiella pneumoniae* and *B*. *cepacia* (Fig. S6). To further examine the pathogen clustering patterns based on pathogenicity and phylogeny, we selected the top 15 most frequent pathogens from Dataset 2 and constructed two trees: (1) an approximately-maximum-likelihood phylogenetic tree; and (2) a hierarchical clustering tree using their Manhattan distances derived from VF SHAP profiles (Fig. 4c). Comparative analysis revealed partial similarity between the two trees, e.g., close clustering of *Listeria innocua* [31] and *Listeria monocytogenes*, as well as *Clostridioides difficile* and *Clostridium perfringens* in both trees. These results highlight the discrepancy between pathogenicity-based and phylogeny-based distance among pathogens.

### 3.4 Pathogen distribution in topsoils across China based on VF-KNN

**Fig. 5:**
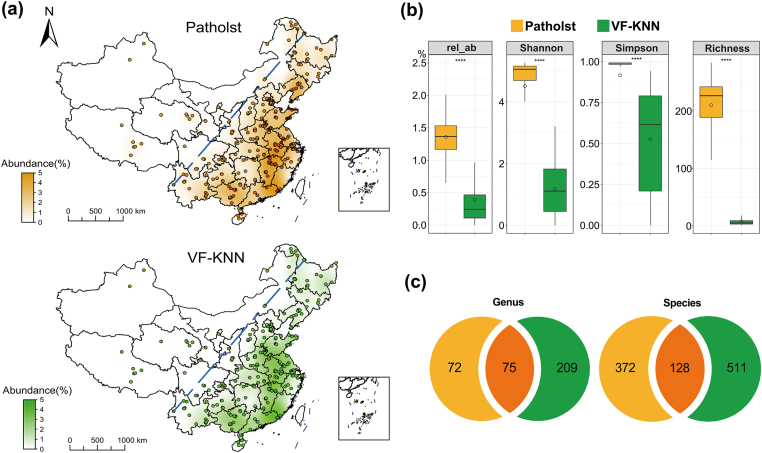
Comparisons between the VF-KNN and Patholst methods in pathogen analysis based on topsoil metagenomes in China. (a) Geographical distribution of pathogens; (b) Overall pathogen abundance and diversity of the two methods (significance level: ∗∗∗∗*p* ​≤ ​0.0001, ∗∗∗*p* ​≤ ​0.001); (c) Number of pathogens identified by the two methods at genus and species levels.

We employed VF-KNN for analyzing the pathogenic bacteria in topsoils across China based on metagenomic data (*n* ​= ​336, including 5 land types). The geographic distribution of pathogens was evaluated (Fig. 5a), and the results were compared with those obtained by the conventional method based on a predefined pathogen list (Patholst).

The two methods differ significantly in overall pathogen diversity and relative abundance. The averaged total pathogen abundance obtained by VF-KNN (0.44%) was lower than that obtained by Patholst (1.4%, *p* ​< ​0.001, Fig. 5b). The abundance of pathogens was higher in the southeast of the Hu Huanyong Line compared to the northwest of the line (0.44% vs. 0.38%), which aligned closely with the levels of economic and social development in these regions. This overall geographical distribution pattern was identical for the two methods. Pathogen hotspots were predominantly concentrated in the eastern coastal provinces, with the highest relative abundance reaching 2.8% (an agricultural land sample).

The alpha diversity of pathogens identified by VF-KNN, as measured by Shannon, Simpson, and Richness indices, was significantly lower than that identified by Patholst (*p* ​< ​0.0001, Fig. 5b). At the genus level, 75 pathogenic genera were identified by both methods, while a substantially larger fraction (209 genera) were uniquely detected by VF-KNN (Fig. 5c). Similarly, at the species level, the total number of pathogens identified by VF-KNN was 28% higher than that identified by Patholst. VF-KNN identified fewer but significantly different pathogenic bacterial species across samples (Fig. 5a), likely due to the strong heterogeneity of soil communities, resulting in a higher total number of pathogenic bacteria than Patholst (Fig. 5c). Patholst, in comparison, identified more but similar pathogenic bacteria across different samples (Fig. 5a). Therefore, the richness values for Patholst were higher than VF-KNN.

Among the pathogens uniquely detected by VF-KNN, there were several less common or understudied species, such as *Mycolicibacterium cosmeticum*, which has recently been linked to indolent, persistent bacteremia [32]. For the 372 pathogenic bacteria identified by the Patholst method but not detected by VF-KNN, this may be due to the excessively low abundance of these pathogens, making it impossible to identify sufficient virulence genes using VF-KNN (approximately 96% of the 372 pathogenic bacteria had an abundance below 0.01%).

**Fig. 6:**
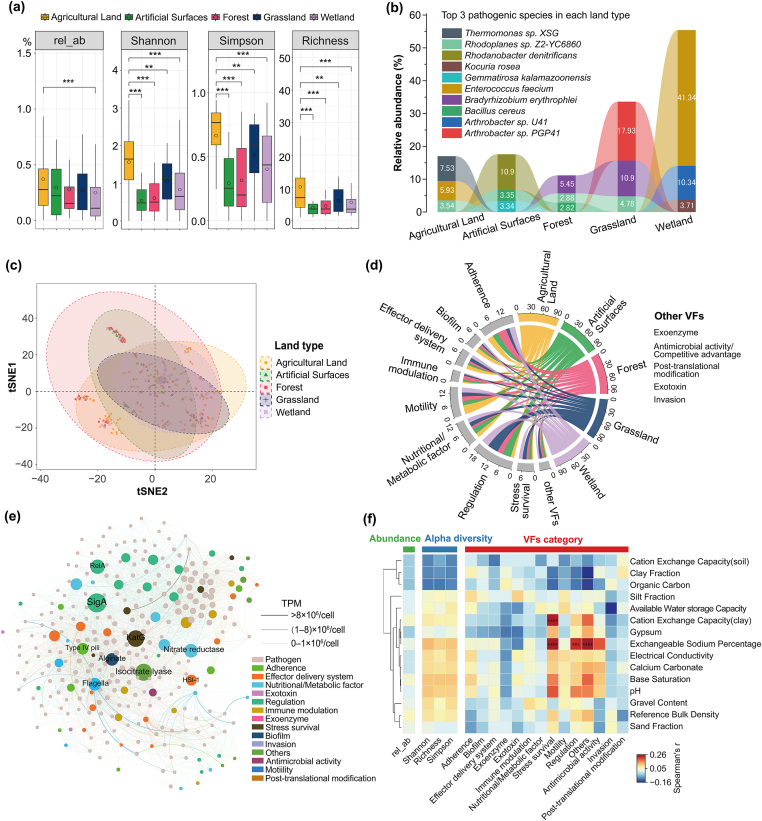
Bacterial pathogens in topsoils across China analyzed by VF-KNN method. (a) Alpha diversity and abundance of pathogens in each land type; (b) Top 3 pathogenic bacterial species and their relative abundance in total pathogens in each land type; (c) Pathogen beta diversity analysis by tSNE based on Bray–Curtis distance; (d) VFs distribution in different land types; (e) Co-occurrence network of pathogens and VFs. The graph was plotted using Gephi. Node size indicates connectivity (degree) between VFs and pathogens. Only VFs and pathogens with a degree ≥10 were plotted. The width of edges represents the TPM values of VFs in specific pathogens. Edge color is in line with the color of VF; (f) Spearman's correlations between pathogen relative abundance, diversity, VFs, and soil properties. Asterisks represent moderate (*r* ​> ​0.2) and significant correlations (∗∗∗∗*p* ​≤ ​0.0001, ∗∗∗*p* ​≤ ​0.001).

Pathogenic bacteria also differed significantly in soils with different land types. Agricultural land possesses the highest relative abundances of pathogenic bacteria, followed by artificial surfaces, forest, grassland, and wetland (Fig. 6a). Notably, the predominant pathogens in each land type vary significantly. In agricultural land, the top 3 pathogens are *Thermomonas* sp. *XSG, Enterococcus faecium,* and *Rhodoplanes* sp. *Z2-YC6860*, accounting for about 17% of the total pathogens (Fig. 6b). *E*. *faecium* is also among the top 3 pathogens in the wetland. They are commonly found in the gastrointestinal tracts of humans and animals, and their prevalence in agricultural land and wetland is likely due to fecal contamination.

In Fig. 6b, the top three pathogenic species identified in each land type have been isolated from soil, water, and air environments, where they actively participate in biogeochemical cycling [33]. Although the vast majority of these microorganisms are not reported to be associated with human pathogenicity, a small number of recent studies have confirmed that certain typically non-pathogenic species can exhibit pathogenic potential under specific conditions. For example, *Rhodoplanes* sp. could be an emerging human pathogen involved in febrile conditions caused by local infection [34]. *Kocuria* sp. are known to be opportunistic pathogens that cause infections in humans, especially immunocompromised hosts [35]. *Arthrobacter* sp. has been clinically associated with infections, including documented cases of bacteremia [36,37]. Notably, for some of the potential pathogenic bacteria, their VFs may pose severe risks to the host. For example, *Gemmatirosa kalamazoonensis* contains alginate virulence factors (contributing to bacterial persistence in cystic fibrosis lungs [38]), and LPS virulence factors (may trigger inflammatory responses [39]).

Significant variations in pathogen diversity were observed across various land types (Fig. 6a). Agricultural land exhibited the highest alpha diversity, whereas no significant differences were found among the remaining four land types. Based on the PCoA analysis (Fig. 6c), significant variability was found among samples both within and between land types.

We further analyzed the VF distribution in different land types (Fig. 6d) and the virulence distribution characteristics (Fig. 6e). Regulation was the most abundant VF category (1.9%–6.2% of the total VFs), followed by Adherence (average: 3.4%). It shows that SigA (Regulation), RelA (Regulation), Flagella (Motility), KatG (Stress survival), Type IV pili (Adherence), nitrate reductase (Nutrition/Metabolism), and HSI (Effector delivery) have a high degree in the VF-pathogen co-occurrence network, indicating their high occurrence in soil pathogens.

Correlation analysis of pathogenic bacteria abundance, diversity, major VFs, and soil physicochemical properties was conducted (Fig. 6f). Stress survival and regulation-related VFs display weak positive correlations with ESP and CEC. This suggests that these soil properties may create conditions that favor the expression or persistence of particular virulence mechanisms. There were limited correlations between total pathogen abundance, alpha diversity, and soil properties. Therefore, the overall presence and diversity of pathogenic bacteria in soil may be influenced more by factors other than the measured physicochemical properties, e.g., microbial interactions in soil communities.

## 4 Discussion

### 4.1 VF-KNN advances pathogen identification based on the metagenome

Traditional pathogen identification methods have several limitations, including the time-consuming nature of genome assembly and potential misclassification and omission due to species annotation-based comparisons with known pathogens. The VF-KNN method addresses these limitations by leveraging the distinct features of VFs between pathogens and non-pathogens. This approach eliminates the need for genome assembly and enables the identification of potential pathogenic bacteria by integrating taxonomic information with VF features.

VF-KNN offers unique advantages in the identification of potential pathogenic bacteria based on metagenomes, providing a robust alternative to traditional approaches. It achieves: 1) high accuracy: the overall pathogen prediction accuracy is about 85%; 2) high sensitivity: the prediction accuracy is maintained around 90% even at low genome fold coverage of 0.4X, achieving accurate identification of low-abundance pathogenic bacteria in the metagenome; 3) ability to identify potential pathogens that are absent in the predefined pathogen list. 511 potential pathogenic species (Fig. 5c) were identified by VF-KNN but not included in Patholst.

The advantages of VF-KNN likely stem from the feature engineering and machine learning framework applied to VF features. Pathogens and non-pathogens exhibit distinct VF features [21,40]. We constructed comprehensive VF feature tables including the total TPM/cell values for category-summed and individual VFs. The VF features included the TPM/cell values of total VFs, category-summarized (14 major categories) VFs, and individual VFs (565 types). It predicts pathogenicity of a query genome by identifying the most frequent class (pathogen/non-pathogen) among its K-nearest neighbors, and employs the Manhattan distance as a similarity metric. Unlike Euclidean distance, Manhattan distance measures the cumulative path length along coordinate axes, making it more suitable for capturing the incremental contributions of VF features to pathogen identification.

PaPrBaG relies solely on k-mer and bacterial protein features for pathogen identification, without incorporating virulence factor characteristics. Additionally, this method uses a random forest model, which lacks biological interpretability due to its ensemble of decision trees. It also incurs high computational costs and long prediction times for large-scale datasets. DCiPatho method employs a deep cross-fusion approach incorporating cross, residual, and deep neural networks, and predicts pathogens based on k-mer features. Similarly, it does not adequately account for the critical role of virulence characteristics in pathogen prediction. Furthermore, the complex fusion neural network makes it challenging to achieve clear explanations of the decision-making process.

In sum, compared to DCiPatho and PaPrBaG, VF-KNN utilizes virulence gene composition as features rather than k-mers, and the KNN algorithm provides clear classification with better interpretability. As a method based on reads and virulence features, we consider that VF-KNN provides a more transparent and interpretable process for pathogen identification. VF-KNN also demonstrates desired overall sensitivity and prediction accuracy, particularly for pathogens in soils. Enhancing the robustness of VF-KNN to detect low-abundance potential pathogens in the environment and improve prediction accuracy remains an important direction for future research.

### 4.2 Characteristics of pathogenic bacteria in topsoils across China

VF-KNN demonstrated superior performance by identifying a greater number of pathogenic bacteria at both the genus and species levels, compared to the conventional method, Patholst. Notably, VF-KNN detected less commonly reported pathogens that were absent in the reference pathogen list, such as *M*. *cosmeticum* associated with immunocompromised states and bacteremia [32], and *Kocuria rosea* associated with bacteremia [41]. These findings underscore the enhanced capability of the VF-KNN method, which leverages VF features to accurately assess bacterial pathogenicity. In contrast, Patholst's reliance on a predefined pathogen list limits its ability to identify emerging or conditionally pathogenic bacteria. This advantage of VF-KNN is particularly critical for identifying pathogens in complex environments such as soil.

Pathogenic bacteria show a notably higher prevalence in the southeast of the Hu Huanyong Line in China. This region, characterized by higher precipitation, warmer temperatures, and more intensive human activities (e.g., agriculture, urbanization), likely creates favorable conditions for the proliferation of pathogenic bacteria. This aligns with previous studies highlighting the complex influences of climatic, environmental, and anthropogenic factors on microbial communities [42,43]. However, there were very limited correlations between the alpha diversity of pathogenic bacteria and a multitude of soil properties. Meanwhile, VFs related to stress survival are positively correlated with ESP and CEC, etc. This is likely because stress survival VFs could enhance resilience to osmotic stress in soils. Overall, the lack of strong correlations between soil properties, pathogen diversity, and abundance could be attributed to the complex interplay of factors, including but not limited to climate, land use, and microbial interactions.

Significant differences exist between agricultural land and other land types in pathogen abundance and diversity. In agricultural land, multiple factors, such as crops [44], soil properties [43], fertilizers [45], irrigation water [46], and climate [47], interact in a complex manner, leading to a highly intricate soil environment. As a result, the abundances and diversities of pathogenic bacteria in agricultural soil are relatively high.

Some of the VF-KNN-identified potential human pathogens in soils could cause severe harm. For example, *E*. *faecium* has been identified in multiple continents with increasing prevalence, which caused up to 250,000 deaths globally in 2019 [48]. *Bacillus cereus,* commonly found in animal feed and food chains, poses risks to the animal industry and human health [49]. *E*. *faecium* was abundant in agricultural land and wetland. This could potentially be attributed to the application of manure and the active degradation of organic matter in these environments, providing sufficient substrates for growth. The abundance of *B*. *cereus* is relatively high in forest soils, which may be related to the higher organic matter content in forests [50]. Additionally, the forest environment presents complex factors under which *B*. *cereus* can enhance its survival probability by producing endospores and forming biofilms [51].

Although the high-abundance potential pathogens identified by VF-KNN are generally considered non-pathogenic, several recent reports suggest that some members may have the capacity to infect humans. For example, *Rhodoplanes* sp. could be an emerging human pathogen involved in febrile conditions caused by local infection [34]. Similar cases have also been reported multiple times in the past. For example, *E*. *coli* can cause diarrheal disease or urinary tract infections only when it breaches ecological barriers or overwhelms host defenses. *Clostridium difficile* was previously considered merely a member of the normal gut microbiota, or its pathogenicity was underestimated. Now, it is clearly identified as the main pathogen of antibiotic-associated diarrhea and pseudomembranous colitis [52]. We acknowledge that further experimental validation is required to confirm the actual pathogenicity of the potential pathogens identified by VF-KNN, particularly for those not overlapping with traditional databases.

On one hand, due to mutations and horizontal gene transfer, non-pathogenic bacteria can acquire virulence genes and become pathogenic. For example, microbiome dysbiosis and host immune deficiencies can transform *E*. *faecium* from a nearly harmless member of the gut microbiome into a cause of bloodstream infections [53]. Horizontal gene transfer events have rendered a prophage-encoded toxin as the primary pathogenic factor in enterohemorrhagic *E*. *coli* or *Corynebacterium diphtheriae* [54].

On the other hand, pathogenicity depends on specific transmission routes and host–microbe interactions. For example, *E*. *coli* can cause diarrheal disease or urinary tract infections only when it breaches ecological barriers or overwhelms host defenses. Further experimental validation is required to confirm the actual pathogenicity of the potential pathogens identified by VF-KNN, particularly for those not overlapping with traditional databases. Whether these potential pathogens can indeed cause human infectious diseases, and under what conditions this might occur, requires further in-depth investigation in the future.

The most abundant VFs harbored by pathogenic bacteria in these land types are predominantly Regulation, Adherence, Motility, Nutrition/Metabolic factor, and Effector delivery system, These VFs determine pathogenicity through the metabolism and regulation of pathogenic bacteria, the transport of effector proteins [55], immune evasion, and colonization [56]. The high centrality of SigA, RelA, Flagella, nitrate reductase, HSI, Type IV pili, and KatG in the co-occurrence network reflects their critical roles in pathogen survival, virulence, and environmental adaptation. Their interconnected functions make them essential hubs in the network, influencing multiple pathways and processes that contribute to pathogenicity. This is in line with their strong impacts on pathogen identification in VF-KNN (high SHAP values).

## 5 Conclusion

We developed a novel pathogen identification method based on virulence factors and the KNN algorithm, which is suitable for identifying pathogenic bacteria in environmental metagenomes with high accuracy, sensitivity, and biological interpretability. Using this method, the geographic pattern of bacterial pathogens in topsoils across China was depicted, where areas with relatively high pathogen risks were along the southeast coast. The method is applicable not only to soils but also to diverse environmental metagenomes. Notably, there are certain limitations to the VF-KNN method, e.g., many metagenomic reads cannot be efficiently utilized in taxonomy assignment by MetaPhlAn, and relying solely on VF features for machine learning may not capture the full complexity of differentiation patterns between pathogens and non-pathogens. In the future, the development of reads-based species identification tools and a better understanding of pathogenic mechanisms of environmental pathogens will further enhance the sensitivity and accuracy of the VF-KNN method.

## CRediT authorship contribution statement

**Shengchun Qi:** Writing – review & editing, Writing – original draft, Visualization, Validation, Software, Methodology, Investigation, Formal analysis, Data curation, Conceptualization. **Shuyan Wang:** Writing – review & editing, Investigation, Data curation. **Yu Xia:** Supervision, Methodology. **Songcan Chen:** Writing – review & editing, Supervision, Methodology. **Huijie Lu:** Writing – review & editing, Funding acquisition.

## Code availability

The code used in this study can be accessed at https://github.com/luhuijielab/VFKNN.

## Declaration of competing interests

The authors declare that they have no known competing financial interests or personal relationships that could have appeared to influence the work reported in this paper.
