# Supplementary material for: Identification of human pathogens in soil by virulence gene-based machine learning method
Source: Eco Environ Health. 2025 Jul 24;4(3):100171. doi: 10.1016/j.eehl.2025.100171 (PMC12355066; doi:10.1016/j.eehl.2025.100171)
Supplement: Multimedia component 1 [file mmc1.docx]

**SUPPLEMENTARY INFORMATION**

**Identification of human pathogens in soil by virulence gene-based machine learning method**

Shengchun Qi ^a^, Shuyan Wang ^a^, Yu Xia ^b^, Songcan Chen^a,c^, Huijie Lu^a,c,*^

^a^ State Key Laboratory of Soil Pollution Control and Safety, Zhejiang University, Hangzhou 310058, China

^b^ Laboratory of Environmental Microbiology and Ecological Genomics, College of Environmental Science and Engineering, Southern University of Science and Technology, Shenzhen 518055, China

^c^ Key Laboratory of Environment Remediation and Ecological Health, Ministry of Education, College of Environmental Resource Sciences, Zhejiang University, Hangzhou 310058, China

* Corresponding author.

E-mail: [luhuijie@zju.edu.cn](mailto:luhuijie@zju.edu.cn) (H. Lu)


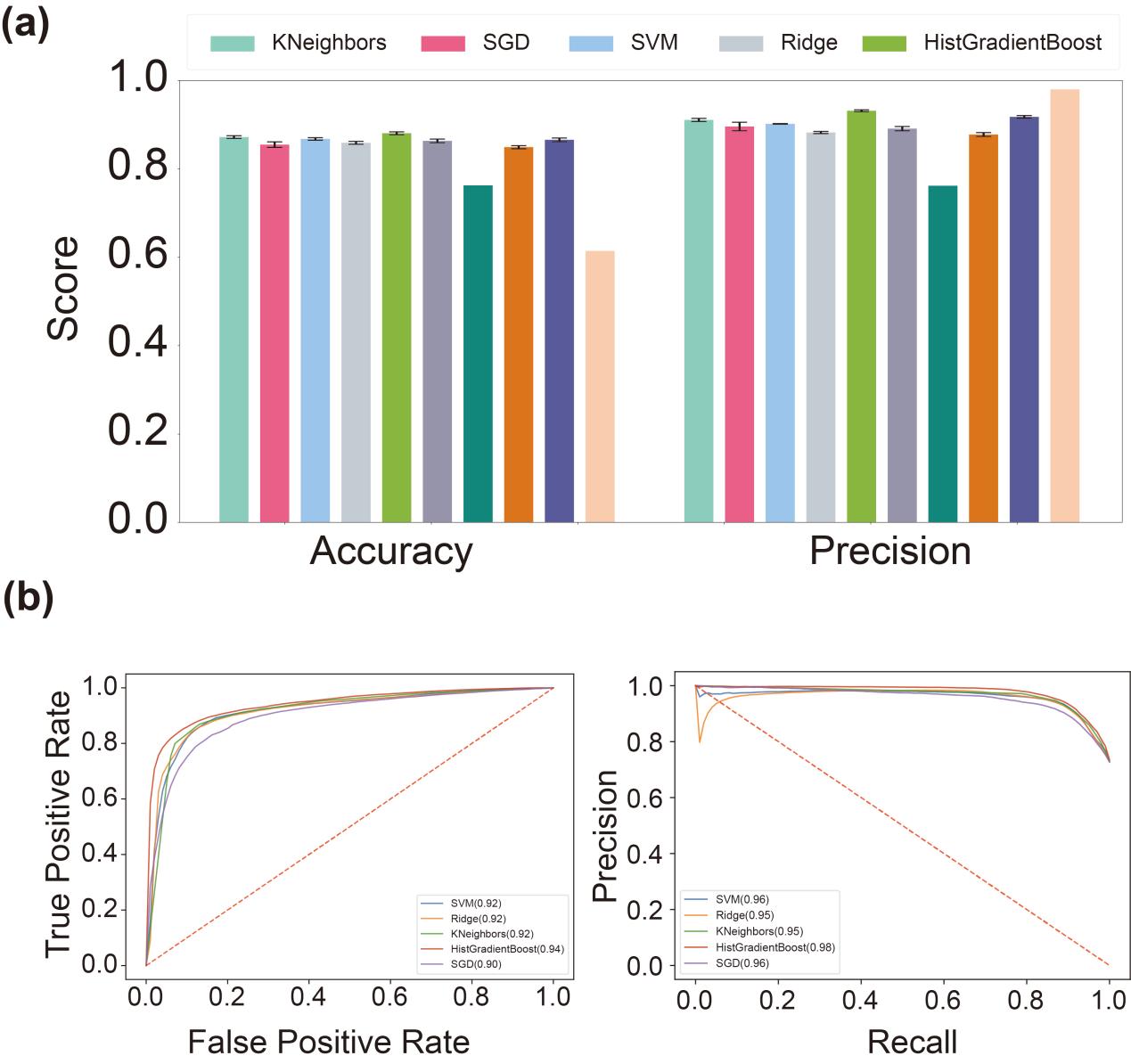


**Figure S1.** Additional performance indices of different models on dataset 1. a) Accuracy and precision scores of 5 machine learning models; b) ROC and PRC curves of the five models.


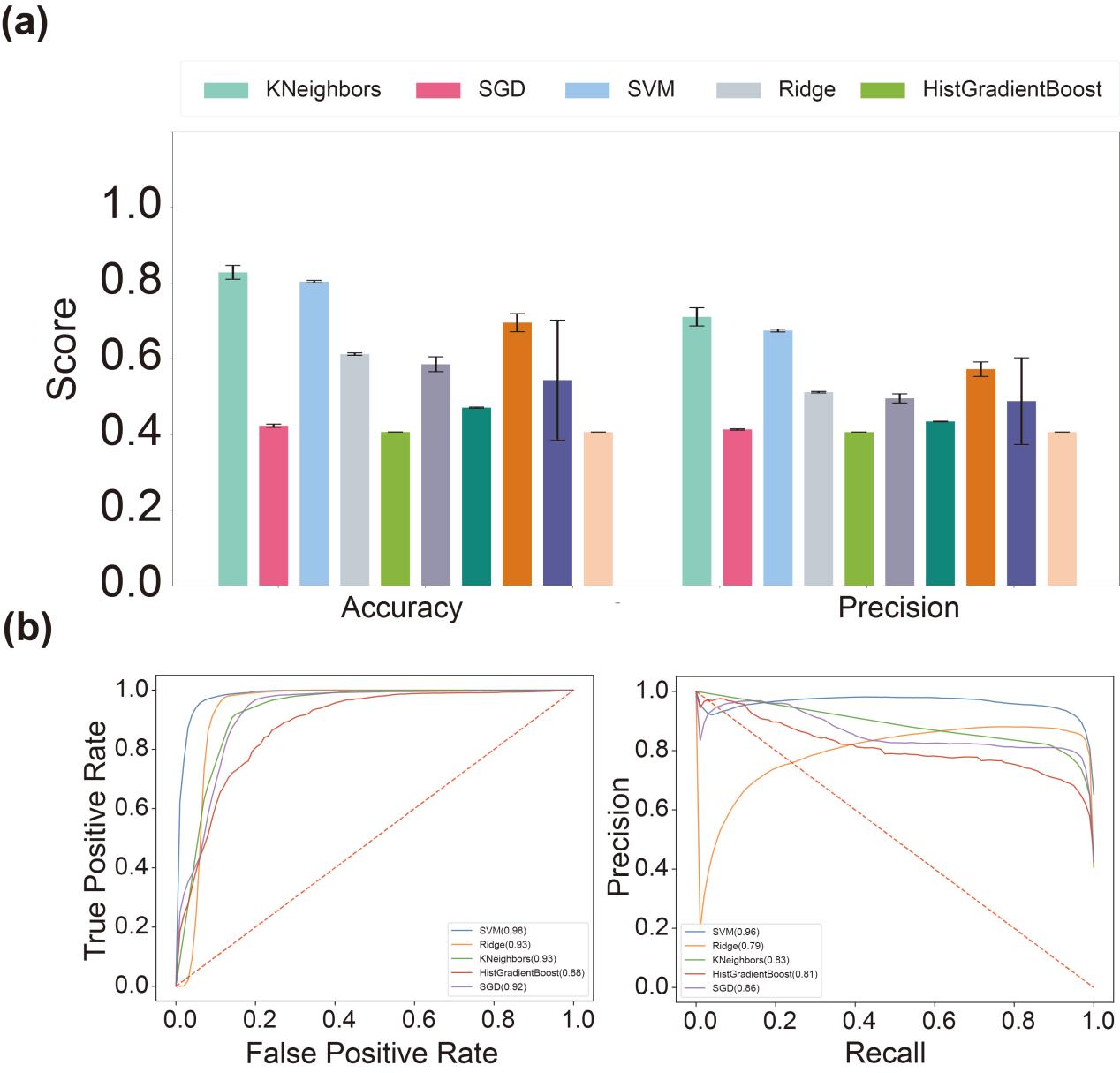


**Figure S2.** Additional performance indices of different models on dataset 2. a) Accuracy and precision scores of 5 machine learning models; b) ROC and PRC curves of the five models.

**Table S1.** Information of 61 pathogens isolated from soil and the pathogen identification results by VF-KNN

| **Number** | **Pathogenic bacteria** | **Isolation source** | **Pathogen (1) or non-pathogen (0) by VF-KNN** |
| --- | --- | --- | --- |
| SY_A_b_1 | *Acinetobacter baumannii* | soil | 1 |
| SY_A_b_2 | *Acinetobacter baumannii* | soil | 1 |
| SY_A_m_1 | *Achromobacter mucicolens* | soil | 1 |
| SY_A_x_1 | *Achromobacter xylosoxidans* | soil | 1 |
| SY_B_c_1 | *Burkholderia contaminans* | soil | 1 |
| SY_B_c_3 | *Burkholderia contaminans* | soil | 1 |
| SY_B_d_1 | *Burkholderia diffusa* | soil | 1 |
| SY_B_d_2 | *Burkholderia diffusa* | soil | 1 |
| SY_b_l_1 | *Burkholderia latens* | soil | 0 |
| SY_B_t_1 | *Burkholderia territorii* | soil | 1 |
| SY_E_ca_1 | *Enterobacter cancerogenus* | soil | 1 |
| SY_E_c_01 | *Escherichia coli* | soil | 1 |
| SY_E_c_02 | *Escherichia coli* | soil | 1 |
| SY_K_a_01 | *Klebsiella aerogenes* | soil | 1 |
| SY_K_p_01 | *Klebsiella pneumoniae* | soil | 1 |
| SY_K_p_02 | *Klebsiella pneumoniae* | soil | 1 |
| SY_P_al_10 | *Pseudomonas alcaligenes* | soil | 1 |
| SY_P_al_11 | *Pseudomonas alcaligenes* | soil | 1 |
| SY_P_al_12 | *Pseudomonas alcaligenes* | soil | 1 |
| SY_P_al_1 | *Pseudomonas alcaligenes* | soil | 1 |
| SY_P_al_2 | *Pseudomonas alcaligenes* | soil | 1 |
| SY_P_al_3 | *Pseudomonas alcaligenes* | soil | 1 |
| SY_P_al_4 | *Pseudomonas alcaligenes* | soil | 1 |
| SY_P_al_5 | *Pseudomonas alcaligenes* | soil | 1 |
| SY_P_al_6 | *Pseudomonas alcaligenes* | soil | 1 |
| SY_P_al_7 | *Pseudomonas alcaligenes* | soil | 1 |
| SY_P_al_8 | *Pseudomonas alcaligenes* | soil | 1 |
| SY_P_al_9 | *Pseudomonas alcaligenes* | soil | 1 |
| SY_P_a_02 | *Pseudomonas aeruginosa* | soil | 1 |
| SY_P_a_10 | *Pseudomonas aeruginosa* | soil | 1 |
| SY_P_a_11 | *Pseudomonas aeruginosa* | soil | 1 |
| SY_P_a_12 | *Pseudomonas aeruginosa* | soil | 1 |
| SY_P_a_6 | *Pseudomonas aeruginosa* | soil | 1 |
| SY_P_a_7 | *Pseudomonas aeruginosa* | soil | 1 |
| SY_P_a_8 | *Pseudomonas aeruginosa* | soil | 1 |
| SY_P_a_9 | *Pseudomonas aeruginosa* | soil | 1 |
| SY_P_o_1 | *Pseudomonas otitidis* | soil | 1 |
| SY_P_p_1 | *Pandoraea pnomenusa* | soil | 1 |
| SY_P_s_1 | *Pandoraea sputorum* | soil | 1 |
| SY_R_m_1 | *Ralstonia mannitolilytica* | soil | 1 |
| SY_St_g_1 | *Stenotrophomonas geniculata* | soil | 1 |
| SY_St_m_10 | *Stenotrophomonas maltophilia* | soil | 1 |
| SY_St_m_11 | *Stenotrophomonas maltophilia* | soil | 1 |
| SY_St_m_1 | *Stenotrophomonas maltophilia* | soil | 1 |
| SY_St_m_2 | *Stenotrophomonas maltophilia* | soil | 0 |
| SY_St_m_3 | *Stenotrophomonas maltophilia* | soil | 1 |
| SY_St_m_4 | *Stenotrophomonas maltophilia* | soil | 1 |
| SY_St_m_5 | *Stenotrophomonas maltophilia* | soil | 1 |
| SY_St_m_6 | *Stenotrophomonas maltophilia* | soil | 1 |
| SY_St_m_7 | *Stenotrophomonas maltophilia* | soil | 1 |
| SY_St_m_8 | *Stenotrophomonas maltophilia* | soil | 0 |
| SY_St_m_9 | *Stenotrophomonas maltophilia* | soil | 1 |
| SY_S_g_01 | *Staphylococcus gallinarum* | soil | 1 |
| SY_S_s_1 | *Stutzerimonas stutzeri* | soil | 1 |
| SY_S_s_2 | *Stutzerimonas stutzeri* | soil | 1 |
| SY_S_s_3 | *Stutzerimonas stutzeri* | soil | 1 |
| SY_S_s_4 | *Stutzerimonas stutzeri* | soil | 1 |
| SY_S_s_5 | *Stutzerimonas stutzeri* | soil | 1 |
| SY_S_s_6 | *Stutzerimonas stutzeri* | soil | 1 |
| SY_S_s_7 | *Stutzerimonas stutzeri* | soil | 1 |

Note: The hemolytic activity of all 61 pathogenic strains has been verified.

Bacterial isolates were obtained from soil samples following a standard protocol. Briefly, soil samples were mixed with PBS (phosphate-buffered saline) and incubated on a shaking incubator at 37 °C for 12 h. After incubation, serial dilutions of the mixture were performed using PBS, and the diluted samples were evenly spread onto selective agar plates specific to different bacterial groups. The plates were then incubated at 37 °C for 12–48 h. Single colonies were picked and subjected to 16S rRNA gene PCR amplification followed by sequencing for species identification. Potential pathogenic isolates were cultured in LB medium for propagation, then stored at −80 °C in 20% glycerol for long-term preservation.

Serotyping was conducted using a combined approach. For bacterial species with available commercial antisera (e.g., *Escherichia coli*, *Klebsiella pneumoniae*, *Pseudomonas aeruginosa*), slide agglutination tests were performed according to standard procedures[1,2]. For species lacking standardized serotyping schemes or antisera (e.g., *Acinetobacter*, *Burkholderia*, *Stenotrophomonas*, *Achromobacter*), in silico serotype prediction was performed based on whole genome sequencing data using SeqSero2 to identify antigen-related loci through genome analysis[3,4].

The dataset of these pathogens are available at https://www.ncbi.nlm.nih.gov/bioproject/?term=PRJNA1264042.

**Details of other benchmark methods**

**DCiPatho:** DCiPatho combines the deep crossfusion of cross, residual and deep neural networks for accurate pathogen detection based on the integrated frequency features of 3-to-7 k-mers. Compared with the existing methods, DCiPatho can accurately identify different pathogenic bacteria that infect humans, animals and plants, it can not only detect known pathogens, but also detect newly emerging unknown pathogens. DCiPatho was tested on dataset2. Paraments were set as default values.

**PaPrBaG:** PaPrBaG overcomes genetic divergence by training on a wide range of species with known pathogenicity phenotype. It remains reliable under very low genomic coverage, this means that it can make effective predictions even when the sequencing data is incomplete or of low quality. PaPrBaG was tested on dataset2. Paraments were set as default values.


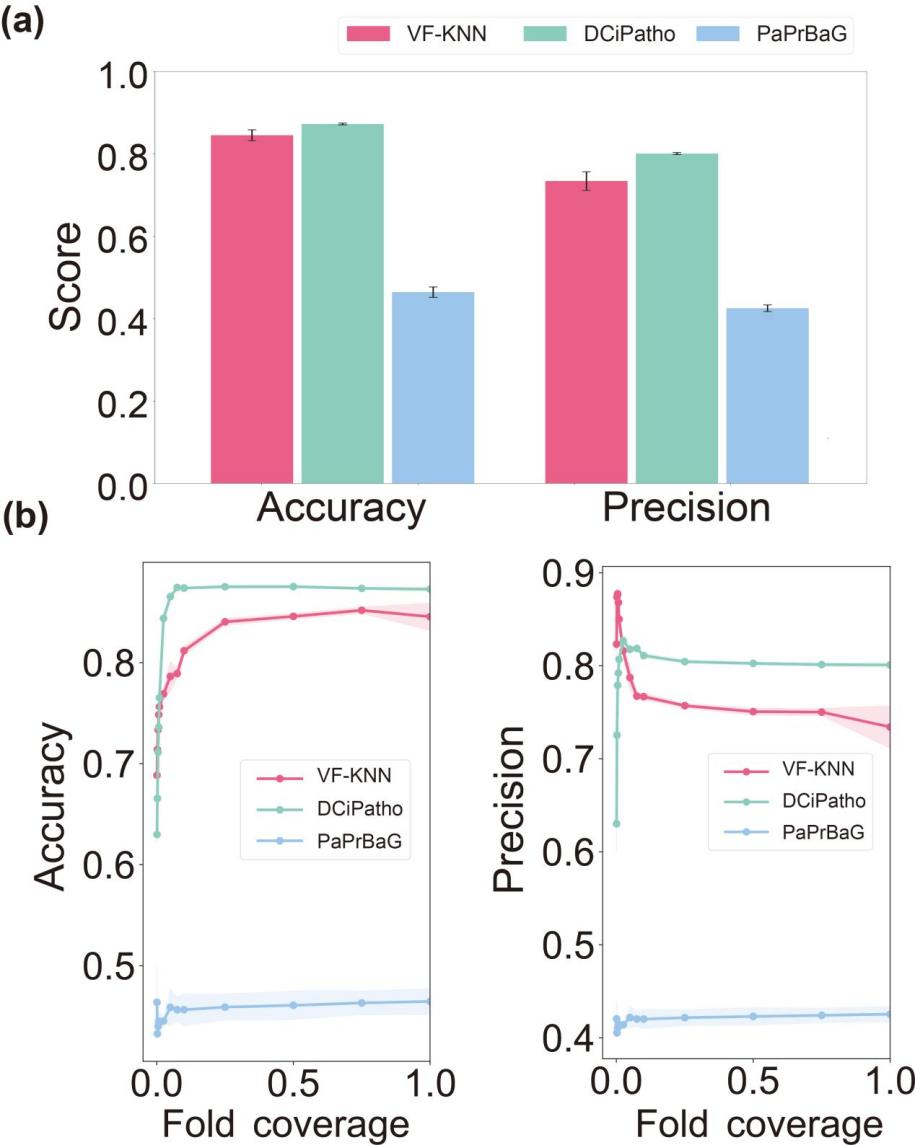


**Figure S3.** Additional performance indices of different methods on dataset 2. a) Accuracy and precision scores; b) Accuracy and precision as a function of genome fold coverage


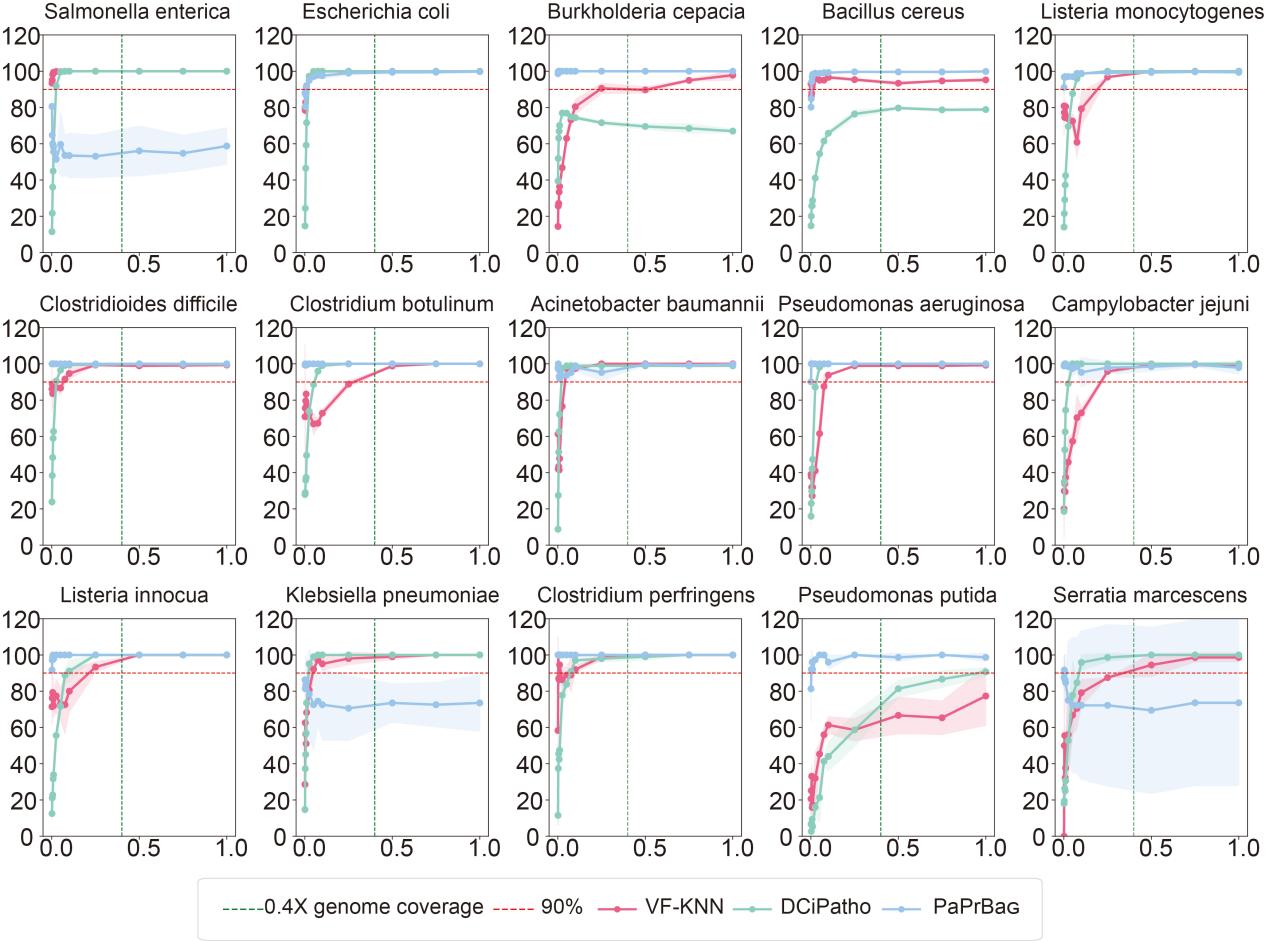


**Figure S4.** Accuracy evaluation on the top 15 frequent pathogens in dataset2 at different fold coverage (0.001X to 1X). x-axis: fold coverage; y-axis: prediction accuracy(true positive rate).

**
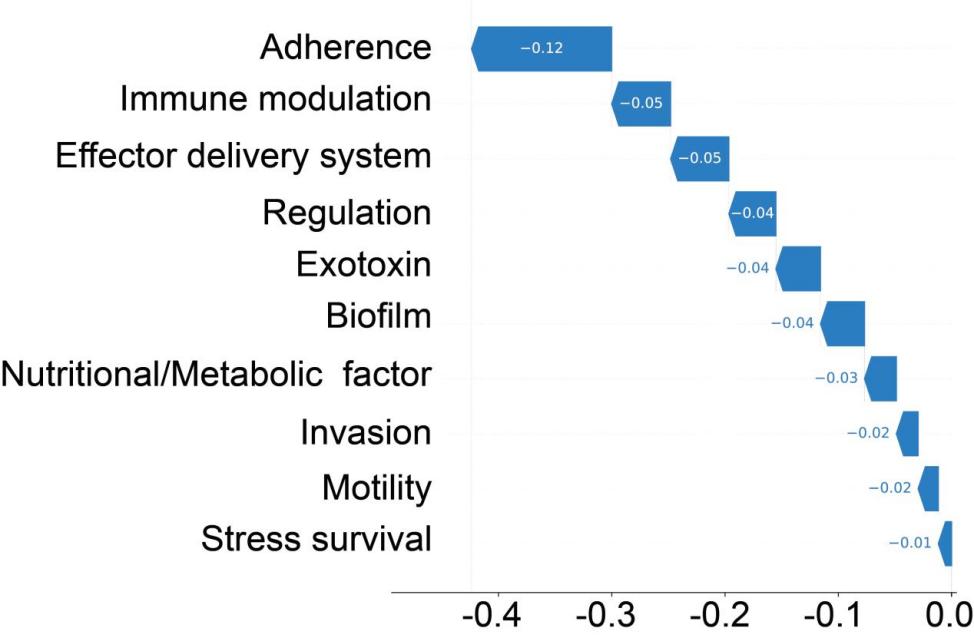
**

**Figure S5.** Mean SHAP values of the top 10 VF categories that impact non-pathogen identification. Numbers are the summed SHAP values of VFs within each category.

**
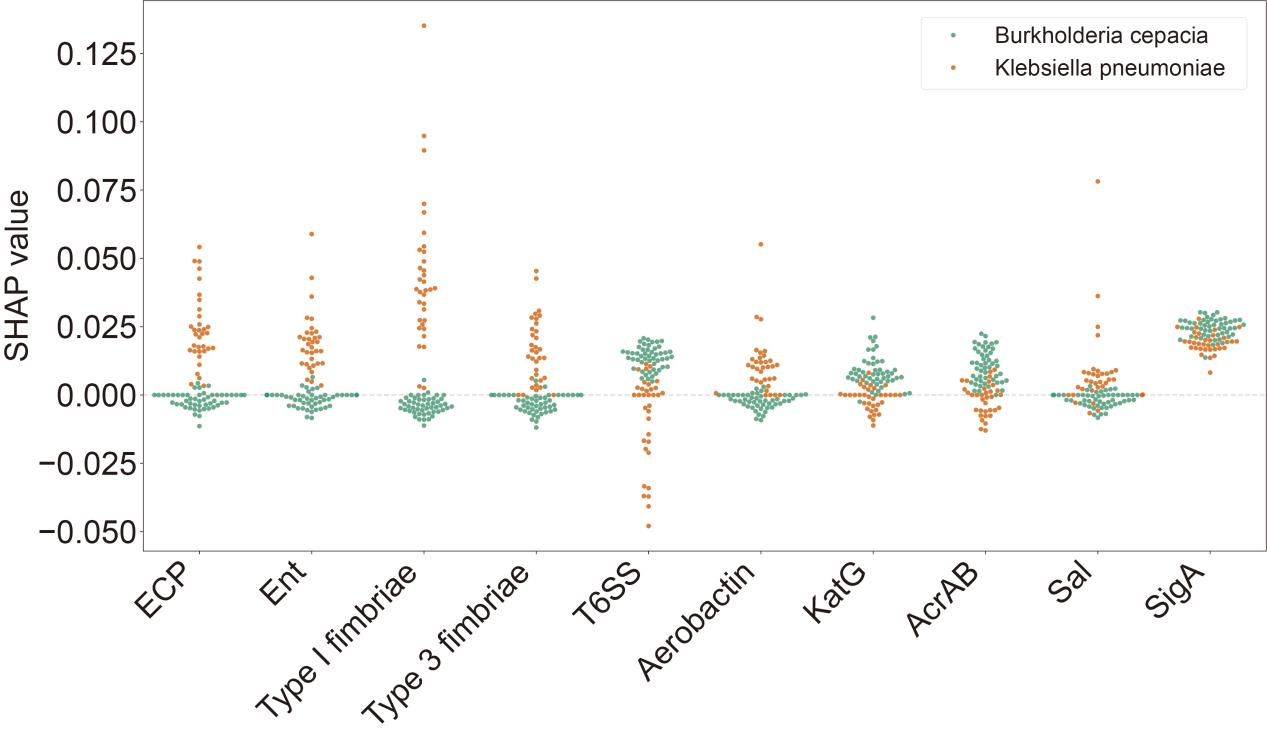
**

**Figure S6.** Detailed SHAP profiles for VFs in *Klebsiella pneumoniae* and *Burkholderia cepacia*

**The computer infrastructure used for model prediction:**

• CPU: 1 X AMD Ryzen 7 5800H with Radeon Graphics

• RAM: 2 X 8 GB DDR4 3200 MHZ

• GPU: 1 X NVIDIA GeForce RTX3050 Laptop GPU(4 GB)

• HD: SSD

• OS: Windows 11 x64


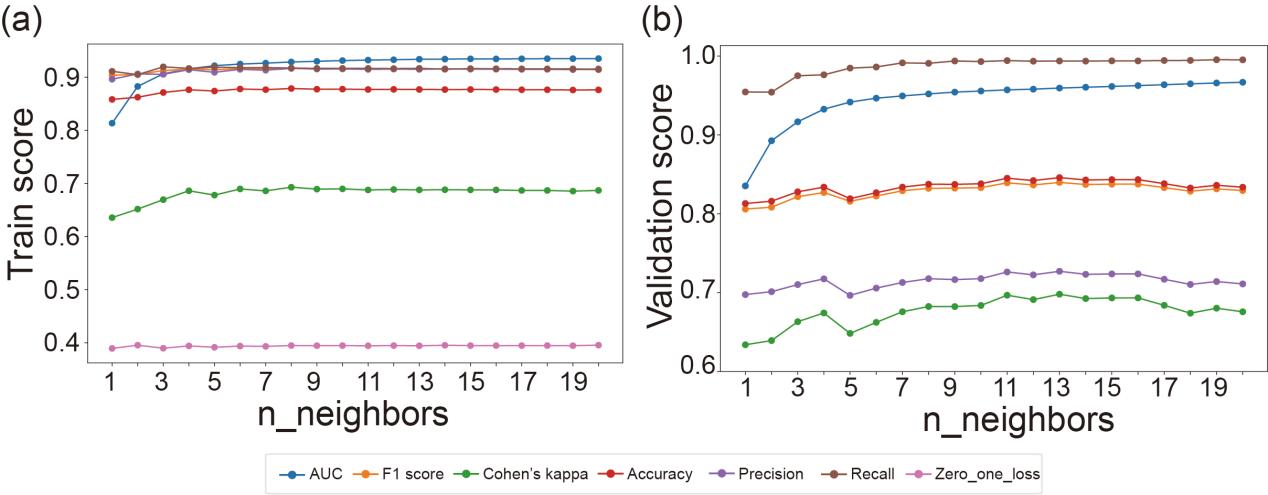


**Figure S7.** Optimization of the n_neighbors in the VF-KNN on training (dataset 1) and validation (dataset 2) data

During feature construction, we scaled features using scikit-learn’s preprocessing function. VF-KNN prioritizes recall, which were superior to the other two methods (Figure 3a), and VF-KNN achieving a false positive rate (FPR) of 0.24—lower than PaPrBaG (0.84) but slightly higher than DCiPatho (0.16). For model optimization, we used empirical risk minimization and five-fold cross-validation. On dataset1, metrics stabilized at n_neighbors > 6; on dataset2, the optimal range for n_neighbors was 8–13. Accordingly, we selected n_neighbors = 10 based on these results (Figure S7).

At 1X genome fold coverage, false positive rate of VF-KNN is approximately 0.24, significantly lower than PaPrBaG's 0.84, though slightly inferior to DCiPatho's 0.16 (Table S2). This suggests that pathogen abundance may be overestimated.

As for the precision and recall values for low-abundance pathogens, after calculation, the precision and recall of the VF-KNN method (Figure 3b, S3) both significantly decrease when genome coverage < 0.1X. This coverage corresponds to a species abundance of approximately 0.001% (with a sequencing volume of 10GB). We consider that for extremely low-abundance pathogens (<0.1X), none of the three models can guarantee ideal precision and recall. Overall, the VF-KNN demonstrates good prediction accuracy for pathogens at genome coverage > 0.4X.

**Table S2.** Comparisons of VF-KNN, DCiPatho, PaPrBaG on dataset2 (1X genome fold coverage)

|  | **VF-KNN** | **DCiPatho** | **PaPrBaG** |
| --- | --- | --- | --- |
| AUC | 0.95 | 0.96 | 0.58 |
| F1 | 0.84 | 0.85 | 0.58 |
| Cohen's Kappa | 0.70 | 0.74 | 0.06 |
| Recall | 0.98 | 0.91 | 0.91 |
| Precision | 0.85 | 0.87 | 0.46 |
| Accuracy | 0.73 | 0.80 | 0.43 |
| False positive rate | 0.24 | 0.16 | 0.84 |
